# Letter V

**Published:** 1805-01-01

**Authors:** 


### LETTER V.

### OF QUACKS AND EMPIRICISM.

THE importance of Dr. Lamert's character had engaged in recital so much space, as to induce me to postpone the history of his eleve, Dr. William Brodum, to the present number, as singly adequate to arrest attention, and acquire admiration; you will therefore now introduce the distinguished person, who should have previously appeared in your valuable pages, under the names of WILLIAM BRODUM, M.D. alias Dr. WILLIAMS; alias

יששבר בר בער הכהך :

alias ISSACHAR BEAR COHEN; alias ISSACHAR the SON of COHEN; constituting CHARACTER V.

The origin of persons, who have risen to great celebrity, has not always been duly recorded in the literary transactions of the times; and, lest future ages should be at a loss, respecting the life, parentage, and education of this successful candidate for fame, we announce that he was born in Copenhagen, the capital of Denmark; in the streets of which he early exercised a public profession, that of hawking and selling ribbons, similar to some of his brethren in London, who dispose of shoe-strings in every avenue near the Royal Exchange.

Far be it from the design of these pages to infer obloquy on the origin of any individual. In a free country like our own, where a full scope is given to the exercise of genius and abilities, many persons, originally obscure, have risen to the brightest ranks in life, and become shining ornaments of society. Many of the chief magistrates of this metropolis, who have filled their stations with dignity, in referring to their line of ancestory, might say with Ulysses,

"Vix ea nostra voco;"

but but whenever distinction has been acquired by laudable and virtuous exertion, it is equally respectable and honourable.

About his twentieth year, young Issachar got his passage to London, and made his first debût on Tower-hill; possessing in his youth a constitution firm enough to enable him to dispense without stockings or shoes, although expert in cleaning the latter for his friends and others, till he was noticed by, and taken into the service of, Dr. Lamert, whom you have already noticed in the 69th Number of your Journal. This event, which laid the foundation of Issachar's fortune, was about the year 1787; at which time, he was employed in going on errands, taking care of Lamert's horse, and officiating in other menial services.

This was long before he assumed the name of Brodum; for at this time, having deserted his own name, as he afterwards abjured his religion, he adopted the name of Dr. Williams, and Lamert kindly equipped him on the occasion from Rosemary Lane; but as he had not arrived to the luxury of duplicates, he kept his bed whilst his nethermost garment was washed by poor Mrs. Bridgmore, who was Lamert's washer-woman, and who kindly procured a lodging in Lamert's neighbourhood, that the boy, Williams, might be within call of his master, now rising into fame and fortune from the sale of Switzer's balsam. Finding Williams a smart active youth, very loquacious, and of sonorous lungs, he procured him boots and spurs, and mounted him á cheval, to circulate far and near the virtues of Switzer and other nostrums; particularly, in the county of Kent and throughout the Isle of Thanet, where his reputation was very generally diffused; but in Hawkhurst, in Kent, and the neighbourhood of Benenden, it became more concentrated and famed; though from what source is not ascertained. A carpenter's widow, however, who had recently lost her husband at Hawkhurst, whose esteem or gratitude for Williams was such, as to induce her to compliment him with a suit of purple clothes and gilt basket buttons. At this town, it is said, an apothecary of the name of Brodum, now deceased, had formerly acquired some reputation, and by the advice of the widow, Williams was transmuted into Brodum, which assumed name he has ever since continued; and to traffic in health,

"*Magni sub nominis umbra.*"

Now metamorphosed into a youth of some fashion and dress, and not void of assurance, Brodum was admitted

 into into a share of Lamert's business, which he not only extended, but procured for himself a considerable degree of personal reputation, and with it consequent emolument.

He did not however appear as a principal on the stage of empiricism, as he had in view the acquisition of a doctor's degree, previously to the assumption of his real character. He represented himself therefore, at this time, as a surgeon, who had served in America with the Hessian army, under General de Heister, during the late war in that hemisphere.

Most Quacks, who assume the title of doctor, possess as fair a claim, with respect to family aud education, as our hero; and though Lamert had no right to this honour, beyond his own presumption, certainly Brodum, under the exercise of vending ribbons in Copenhagen, and the education which the colleges in Rag-fair, near Tower-hill, afforded, could not hence plead superior claim to such a distinction; but his genius, perseverance and cunning surmounted every obstacle, and procured him as substantial a doctor's degree, as a venal Scotch University can confer. To effect his artful project, the assistance of Dr. Leo, a Jew, in the vicinity of St. Mary Axe, was procured.

It is very well known, that in Aberdeen, and St. Andrews, the professors have been in the practice of conferring the degree of doctor of medicine, without the least examination, or personal knowledge of the candidate; but merely on the recommendation of some respectable physician. In these degrees, the persons on whom they are conferred, are uniformly said to be admitted from their great medical knowledge, &c. in the following strain, which is copied from the Marischal College of Aberdeen, "As it has been an ancient and laudable custom, that those who have applied themselves to learning with much labour and assiduous study, should be honoured with some singular mark of distinction, as a testimony of their successful perseverance, and a reward for their extraordinary merit, that the rising generation may be excited by such example to pursue the like arduous but glorious career of erudition and virtue. Therefore we, Alexander Donaldson, with the unanimous consent of the Rector, Principal, and the other Professors of the said University, do create, declare, and appoint the above William Brodum, Doctor of Physic."

The only difficulty, therefore, in the way of Brodum's acquiring all these meretricious praises, was to procure a recommendation to one of these Universities; to effect which, which, he made this Dr. Leo the tool to carry on his impostures; who, in his name, applied to an eminent Physician and Fellow of the Royal College of Physicians in London; and sounded such an eulogy in favour of Brodum, as procured from this physician a recommendation to Aberdeen; and a doctor's degree was the result. This was certainly prior to Brodum's assuming the public character of an empiric; but as soon as he had acquired this honour, he threw off the mask, and appeared in his own character of a quack doctor, screened by a degree acquired by falsehood and deceit; and which he has ever since impudently published with his respective quack bills.

The moment that the professors of Aberdeen were apprized of this deception, and their consequent degradation, they applied to the eminent Collegian who had recommended Brodum to their patronage, not without some reproaches for the degradation this unguarded step had entailed. He replied with that candour and sincerity with which his character has been uniformly distinguished; and fully explained to them, the arts employed to deceive him; and added, that he was ready, if they requested it, to make a public avowal of the whole base imposition; at the same time suggesting, that as the degree had been conferred, whether it were not most prudent to pass it over in silence. Unfortunately, the professors adopted this suggestion, and Brodum continues to puff himself off, as a learned graduate of the University of Aberdeen; a place he never saw, nor have the professors ever thrown their eyes over their *learned* associate.

I am however bold to assert, that the college continues accesory to its own degradation; for the moment that the imposition was discovered, they ought to have rent asunder the surreptitious veil, and exposed to the world the impostor. If, under their sanction, he rises, and indeed he has risen, into celebrity, and scatters injudicious nostrums amongst his votaries, the professors are amenable to the unerring tribunal of Heaven. If their own laws, or the just rights of men, do not rouze them from their apathy, to support dignity of character, and excite them to rescind an act surreptitiously procured under a fictitious name, involving in its operation the health and happiness of the community, they deserve all the infamy consequent on sanctioning the base traffic in human constitutions. Every literary society, which acknowledges laws for the admission of members, ought to possess the salutary

 ones ones of expulsion; and, doubtless, every public body claims these salutary powers, without which society could not be safely maintained. To hesitate upon the conduct that ought to have been adopted, in the imperious alternative of life or death, evinces equal depravity and meanness. It is as honourable to confess as it is infamous to maintain an error; and in the name of insulted humanity, I call upon the professors of Aberdeen, to restore the dignity of their degraded University, and no longer to suffer their names, published to the whole world in capital characters, to remain as the abetters of a class of beings, who sport with health for lucre. They should instantly tear off the honours conferred on a notorious impostor, and return him the thirteen guineas received as the price of their dishonour, as a just atonement for error; thereby emulating the conduct of Judas Iscariot, whose remorse impelled him to restore the money he had accepted as the price of blood; unless, like the witch of Endor, they can conjure up the living from the dead, who have been immolated on the altar of ignorance and unblushing impudence, which they have erected by their suffrages, and sanctioned by their names.

Under this high and imposing authority, Dr. Brodum rapidly rose to fame and fortune. Although totally ignorant of medicine, he had acquired the science of knowing the weakness of human nature, and upon this he conducted his operations. He well knew that in a wealthy luxurious metropolis, where the indulgence of vicious propensities would entail corporeal debility, that a medicine which promised restorative powers, must become popular, both with debilitated youth and worn-out age; and he therefore seductively entitled his remedy, his "Nervous Cordial;" to which he added his "Botanical Syrup for debilitated constitutions," with a "Guide to Old Age," impudently dedicated to the King's Most Excellent Majesty, with "a portrait of the author."

This medicine was chiefly the old formula of the decoction of the woods, consisting of sassafras, guaiacum, and a few other articles, which he procured of Mr. Chamberlin, an eminent chemist in Fleet Street. The decoction is well edulcorated with sugar or molasses, and sold at six shillings and sixpence a bottle, or one pound two shillings for five bottles.*

If the College of Aberdeen, distinguished by a Beattie who charmed and instructed mankind by his *Minstrel*, and by his "*Immutability of Truth*" dare to espouse and promote promote ignorance and imposture by a public falsehood, what avails erudition? The Professors declare, in the face of the world, that they have *unanimously conferred a Degree on William Brodum, and publicly recommend him as an example to the rising generation, to pursue his arduous but glorious career*, although he never entered a College, University, or School of Medicine. If his career be *glorious*, why do these very Professors not only give public lectures, but insist upon the necessity of the attendance of pupils upon them for a series of years, in order to qualify them for the discharge of their professional duties; whilst *they recommend William Brodum as an example to the rising generation?* They ought, consistently, to close their lectures, and shut up their colleges, and turn them into laboratories for the sale of quack medicines. Oxford and Cambridge might follow the laudable example; and that school, where thousands listened with rapture to a Monro, a Rutherford, a Gregory, or a Cullen, may become a repository for nervous and restorative cordials, under the auspices of twin Jews, the celebrated Solomon, and no less distinguished Brodum, whose *glorious career* is alone proclaimed by the Professors of Aberdeen.*

In all periods of history when persons, whether from vice or age, are no longer capable of enjoying healthful life, various plans have been suggested and pursued for renovating and preserving it. The blood of young persons or animals has been conveyed into the veins of aged persons, in order to render them younger; and Heister in his Surgery has described this process. In the Philosophical Transactions, there are several examples of the transfusion of blood related, particularly No. xix. p. 352; No. xx. p. 353; by Dr. Lower. No. xx. p. 356; No. xxv. pp. 449, 451.

* As William Brodum is a fictitious or assumed name, the Professors of Aberdeen might avail themselves of this imposition, by recalling and expunging a diploma conferred on a fiction, or nonentity, to which the supposed graduate could not in law or equity be entitled.

The assumed William Brodum was born and educated a Jew, and received the name of Issachar Bear Cohen, or Issachar the son of Cohen; and applying Bear (which means son of) to a crest, he has assumed the bear's head as such, though importing a very different meaning.

William Brodum not being in existence, a diploma conferred on a fiction instantly ceases to operate; it is the law of this country in every misnomer, even to the omission of a letter; and the Marischall College will I trust see the necessity of pursuing a course founded upon law and justice; for Wm. Brodum is no more a Doctor than my house-dog, or the well known man in the moon.

451. In No. xxvi. p. 479, "An old dog almost blind with age, and incapable of much action, did leap and frisk, after receiving the blood of a young dog." This wonderful account is by Mr. Gayaut. Another author (No. xxv. p. 453) declares, that an enfeebled dog was rendered strong and vigorous by receiving the blood of a calf; but I do not find that the dog was otherwise metamorphosed. See No. xxvii. p. 521; No. xxx. p. 559; No. lxii. p. 840; and the French Academy.

In the days of chivalry, a certain Arcadian fountain was sought after for the very desirable purpose of renovating life, and some early navigators thought they had discovered it in America. It is certain, however, that Lewis XI. of France, famous for his mistresses, his bastards, and his devout pilgrimages, shut himself up in the castle of Plessis-les-Tours, and, inaccessible to every one, surrounded by guards, and a prey to the most bitter reflections, sent for a hermit of Calabria, called Francisco Martorillo, since adored as a saint, under the name of Saint Francisco de Paulo; and throwing himself at his feet, intreated him with a flood of tears, to intercede with the Supreme, that his life might be prolonged. Alas! neither Dr. Solomon, nor Dr. Brodum were then in being, to prolong that of others! He was therefore recommended to recruit the weak remains of his life by drinking the blood of young children, fondly imagining to correct thereby the acrimony of his own. This prince always went covered with relics, and constantly wore a leaden figure of the Virgin in his hat; of which he used to ask pardon for his murders before he committed them. In his piety, he made Mary the mother of Jesus, a Countess, by conferring a deed of the Earldom of Boulogne on the Holy Virgin.

To return to the fountain above mentioned, said to make old people young, it was once generally credited. Peter Martyr not only believed it possible, but adduces proofs of it. Vide Dec. ii. ch. 9, p. 93; and particularly Dec. vii. chap. 7, p. 265. It is known, says Bossu in his Travels, vol. ii. p. 6, that Don Juan Ponce de Leon, discovered Florida, as he was in search of Bemini, the isle containing the river Jordan, and the fountain so renowned by the Indians of Cuba; who asserted, that the waters had the quality of making men young again. Leon believed this fable, and went in search of the fountain, but without finding it. He sent Captain Perez de Artubia, and the Pilot de Antonio de Alminos, upon this discovery. He touched at the bay of Puerto Rico, where he found Bemini, mini, but neither the river Jordan, nor the fountain. Don Juan died sometime after, unsuccessfully searching for this famous spring. See Hackluyt's Discoveries, in Oxford's Collection of Voyages, vol. ii. p. 380. At this day, among the Siberians, as was formerly among the Arabians, vivifying liquids for preserving from age are publicly sold. See Gmelin Journ. 1734. Hist. Gener. des Voy. tom. xxiv. p. 189.

When the account of the renovating fountain, we have alluded to, was first transmitted to Madrid, many Spaniards embarked at Cadiz to go in pursuit of it; but when they returned, every one found they had been deceived; instead of being young, they were indubitably grown older, and the people laughed at their long and troublesome voyage; which, however, was attended with the discovery of Cape Corrientes. Upon the coast of India, the women drink a decoction of prime-print to preserve their youth. See Nieuhoff's Travels to the East Indies, Churchill's Collect. vol. ii. p. 330. Hist. Nat. de l'Islande, tom. ii. p. 202, 252. Buffon, Nat. Hist. tom. iii. p. 485.

That Dr. Brodum's botanical syrup of sassafras and sugar is preferable to those remedies I have just enumerated, must be candidly acknowledged, although old age may be as securely protracted by one as the other; and it may be presumed, that it is also more pleasant than the blood even of infants; and high priced as is this syrup, it amounts to much less expense than would a voyage to the renovating fountain of Bemini.

In justice likewise to Dr. Brodum's character, let it be added, that he possesses a disposition to charity, and has liberally opened his purse upon application, though too often he has announced his beneficent actions in the daily prints; once indeed he modestly omitted to publish his donation of twenty-five pounds to the parish he resided in, on account of a proposal at the same time, in a large company at the parish meeting, to drink his health; when Mr. C. a respectable parishioner refused to put this toast, without adding "Lady M. to Dr. Brodum." Mr. C. well knew that the person who proposed the health of Dr. Brodum, had been employed to subscribe "Lady M." to cures that never existed; these and similar puffs being usually dated from watering and other public places, to deceive the credulous. The supposed Lady M. withdrew the motion, and whispered in Mr. C.'s ear, that he was the genuine lady, who earned a *decent* living by forgeries of this *innocent* nature.

A gen-

A gentleman of Dr. Brodum's moral scruples, could not but devote the most pious attention to religion and virtue. That religion which a Goldsmidt, a Solomon, a Sequeira, a Myers, an Eliacon, and other respectable Jews, thought it no dishonour to support and profess, was no longer sufficiently pure for the tender conscience of Dr. Brodum; and he abjured the religion of his forefathers, to support the purity of christianity!

Dr. Brodum has recently visited the place of his nativity, in his route to Berlin, whither he went for the purpose of purchasing the title of Count or Baron. If money would give eclat, and credit secure honour, he travelled with the requisites, carrying in his pocket a letter of credit for fifty thousand rix-dollars, whilst Dr. F. a regular and respectable member of the College of Physicians of London, who met him at Copenhagen, possessed one of an hundred pounds only. A difference and distinction of such magnitude, gained Brodum considerable attentions, and arrested the notice and regard of Dr. Winslow, the son of the great anatomical writer. Thus courted and noticed, Brodum assumed the airs of a great man, and amply confirmed the sentiments of D'Argens.

" Jétois devenu si fier et si vain, que je n'etois plus le fils de mon pêre et de ma mère. *La Richesses* ont la vertu du fleuve Lèthè pour nous faire oblier nos parens et nos amis." Gil Blas, L. viii. ch. 13.

This meretricious parade, however, afforded no effectual passport to the acquisition of a Barony from the Court of Berlin. In the time of Maria Theresa, of Vienna, her valet de chambre acquired a fortune by the sale of titles, which the Empress, in her old age, was dotard enough to sign; the price of a Barony was usually two hundred rix-dollars, and the next inferior title was one hundred; but the Cabinet of Berlin, ever since the period of the great Duke William, has been particularly scrupulous in delegating titles. Frederic the Great was still more guarded, and rarely conferred titular distinction on any but conspicuous statesmen, and distinguished military characters; nor did the present Cabinet deem the feats performed by sassafras and molasses, under the puissant direction even of Dr. Brodum, sufficiently palatable for entailing any honourable claim to nobility. Nor would the abjuration of a religion which a Bloch, a Mendelsohn, and a Marcushertz had professed, plead in his favour. The first, an acute Ichthyologist, had the patronage of the great Frederic; and the learned Mendelsohn, one of the wisest and most moral moral characters then in Europe, possessed his friendship as well as his admiration, whilst his health and life were under the destiny of his physician Marcushertz. In the time even of the late king, when Bishopswender governed his conscience, and Madame de Voss gratified his pleasures, titles of nobility were not "matters that were dealt in by attorneyship," or in one or two instances at the most, where they had been undeservedly conferred, they were abrogated, and the parties imprisoned.

From a consideration of these documents, it may be concluded that Dr. Brodum returned, as to a title of nobility, re infecta. He embraced, however, the most favourable time in his application, when Dr. Brown, the present king's physician, was in England, on a visit to the Duchess of York. On his return he may contemplate with astonishment, the *modesty* of this illustrious protegè of the Marischall College of Aberdeen!

Dr. Brodum, alias Issachar Bear Cohen, prior to his visit to the Continent, disposed of his nostrums, for a very considerable premium, to Mr. Dickinson* of Doctor's Commons; and probably with an ample fortune: though from the mode of remunerating the lawyer, whose opinion he took on the alienation of them to Mr. Dickenson, it might be otherwise suspected. On this occasion, he offerred, not more shillings than formerly he would freely have given guineas; for let it be again remarked, that Dr. Brodum has afforded many laudable instances of pecuniary liberality; he is likewise of a social and convivial disposition, and entertains his friends with occasional songs when called upon in company. His income from vending medicines to make young people strong, and old people young, has been estimated at five thousand pounds per annum; and the most superb carriage in the metropolis, conveys him through its streets, in the midst of a gaping and admiring multitude, amongst whom he has arrested the attention of

IETROS.

---

* Mr. Swinton is now the proprietor, by *purchase*, of Dr. Brodum's Botanical Syrup and Nervous Cordial.

